# Metabolic turnover of cysteine-related thiol compounds at environmentally relevant concentrations by *Geobacter sulfurreducens*

**DOI:** 10.3389/fmicb.2022.1085214

**Published:** 2023-01-11

**Authors:** Mareike Gutensohn, Jeffra K. Schaefer, Torben J. Maas, Ulf Skyllberg, Erik Björn

**Affiliations:** ^1^Department of Chemistry, Umeå University, Umeå, Sweden; ^2^Department of Environmental Sciences, Rutgers University, New Brunswick, NJ, United States; ^3^Institute of Inorganic and Analytical Chemistry, University of Münster, Corrensstraße, Münster, Germany; ^4^Department of Forest Ecology and Management, Swedish University of Agricultural Sciences, Umeå, Sweden

**Keywords:** anaerobe bacteria, low-molecular-mass thiols, cysteine homeostasis, penicillamine formation, *Geobacter sulfurreducens*

## Abstract

Low-molecular-mass (LMM) thiol compounds are known to be important for many biological processes in various organisms but LMM thiols are understudied in anaerobic bacteria. In this work, we examined the production and turnover of nanomolar concentrations of LMM thiols with a chemical structure related to cysteine by the model iron-reducing bacterium *Geobacter sulfurreducens*. Our results show that *G. sulfurreducens* tightly controls the production, excretion and intracellular concentration of thiols depending on cellular growth state and external conditions. The production and cellular export of endogenous cysteine was coupled to the extracellular supply of Fe(II), suggesting that cysteine excretion may play a role in cellular trafficking to iron proteins. Addition of excess exogenous cysteine resulted in a rapid and extensive conversion of cysteine to penicillamine by the cells. Experiments with added isotopically labeled cysteine confirmed that penicillamine was formed by a dimethylation of the C-3 atom of cysteine and not *via* indirect metabolic responses to cysteine exposure. This is the first report of *de novo* metabolic synthesis of this compound. Penicillamine formation increased with external exposure to cysteine but the compound did not accumulate intracellularly, which may suggest that it is part of *G. sulfurreducens*’ metabolic strategy to maintain cysteine homeostasis. Our findings highlight and expand on processes mediating homeostasis of cysteine-like LMM thiols in strict anaerobic bacteria. The formation of penicillamine is particularly noteworthy and this compound warrants more attention in microbial metabolism studies.

## Introduction

Low-molecular-mass (LMM) thiols are an important class of compounds involved in many biological and biogeochemical processes. The thiol (RSH) functional group has several distinct properties including its acidity constant, nucleophilicity, redox properties, and affinity towards metal ions. The critical roles of LMM thiols in maintaining cellular redox potentials, including protection of the cell from reactive oxygen species, and in metal detoxification are well recognized (Jocelyn, 1972; Friedman, 1973; Huxtable, 1986). Recent research has revealed an even more complex picture of the metabolic turnover of thiols in cell metabolism, including their ability to activate virulence (Reniere et al., 2015) or trigger biosynthesis of anti-infective agents (Wang et al., 2015). The metabolism of thiols has predominantly been studied for eukaryote biota and phytoplankton while less is known regarding such processes in bacteria, and in particular anaerobes (Fahey, 2013; Wang et al., 2015). It is clear, however, that the activity of anaerobe microbes contributes to keeping concentrations of LMM thiols in the pM-nM range in anoxic porewaters, highlighting the importance of such microbes for thiol turnover in the environment (Kiene et al., 1990; Kiene, 1991). In this work, we study the metabolic turnover of environmentally relevant concentrations (nM) of LMM thiols by *Geobacter sulfurreducens*, an important iron-reducing bacterium common in sub-surface environments (Methe et al., 2003; Mahadevan et al., 2006).

*G. sulfurreducens* and related bacteria species have been the focus of numerous studies involving bioremediation of groundwater contaminated with organic compounds and metals, production of electricity from organic waste and methylation of inorganic mercury (Hg(II)) to methylmercury (MeHg; Schaefer and Morel, 2009; Lovley, 2012; Knoche et al., 2016). Although the genome, proteome, and metabolism of *G. sulfurreducens* have been well studied (Methe et al., 2003; Ding et al., 2006; Mahadevan et al., 2006), there is a noteworthy knowledge gap regarding the identity and metabolism of LMM thiols associated with *G. sulfurreducens* and with anaerobe bacteria in general (Fahey, 2013). For example, the synthesis of glutathione, which is an important intracellular LMM thiol in many prokaryotes and eukaryotes, is not observed in *G. sulfurreducens* (Wang et al., 2013; Adediran et al., 2019). One earlier study (Adediran et al., 2019) reported production and cellular export of eight different LMM-thiol compounds by *G. sulfurreducens* but it remains unclear what cellular pathways are involved and their biochemical function.

Phytoplankton are known to secrete organic molecules, including thiols, for the acquisition of essential trace metals and to decrease the accidental uptake of toxic trace metals (Dupont et al., 2004; Dupont and Ahner, 2005; Hopkinson and Morel, 2009; Vraspir and Butler, 2009). Much less is known about how bacteria acquire essential metals under anaerobic conditions, and maintain metal homeostasis. The knowledge gaps include the identity and role of LMM thiol compounds in anaerobic *Desulfobacterota* that do not produce thiols (e.g., glutathione and others) characteristic of aerobic bacteria and Eukarya, and if these thiols serve similar functions (Fahey, 2001; Fahey, 2013; Glass et al., 2014). Furthermore, in nature heterotrophic microorganisms like *G. sulfurreducens* are mainly present in complex microbial consortia, biofilms, which often contain mixed autotrophic and heterotrophic communities (Flemming and Wuertz, 2019). Elevated concentrations (400–2000 nM) of LMM thiols have been observed in such biofilms and have been linked to photosynthetically active microbes in some studies (Tang et al., 2000a; Al-Farawati and Van Den Berg, 2001; Dupont et al., 2006; Leclerc et al., 2015) and to dark conditions in others (Bouchet et al., 2018). In addition to their endogenous thiol production and turnover, bacteria like *G. sulfurreducens* may thereby be exposed to enhanced levels of exogenous thiols produced by other organisms.

Thiols, in particular cysteine, have often been added to media of *G. sulfurreducens* assays to serve as an electron shuttler (Kaden et al., 2002), to facilitate dissolution of Fe(III) minerals (Doong and Schink, 2002) or to control the chemical speciation of Hg(II) in methylation studies (Schaefer and Morel, 2009). The fate of added thiols during the course of such experiments has rarely been monitored and thus, the bacterial response to external exposures is uncertain. Sulfide and sulfonate formation has been demonstrated at addition of high concentrations of cysteine (100 μM) in *G. sulfurreducens* assays (Thomas et al., 2018), but the fate of exogenous cysteine at more typical environmental concentrations (nM) is unknown. The metabolism of LMM thiols at such concentrations is largely uncharacterized, not only in *G. sulfurreducens* but in anaerobes in general, and research has been hindered by previous limitations of analytical methods.

Here, we use a recently developed state-of-the-art mass spectrometry-based methodology (Liem-Nguyen et al., 2015) to enable studies of metabolic turnover of LMM thiols at environmentally relevant concentrations in the nM range. Because of the large knowledge gaps at such low thiol concentrations for bacteria in general we take an exploratory approach and focus on a set of LMM thiol compounds with a chemical structure related to that of the amino acid cysteine. Cysteine is an important thiol in many organisms and its structure is present in a number of important intracellular LMM thiols as well as in many proteins (Hand and Honek, 2005; García et al., 2015; Poole, 2015). Cysteine is further a commonly exported LMM thiol by phytoplankton (Dupont and Ahner, 2005) and also by *G. sulfurreducens* (Adediran et al., 2019) and it has been reported prevalent in oxygen deficient environments (Tang et al., 2000b; Zhang et al., 2004; Leclerc et al., 2015; Bouchet et al., 2018; Van et al., 2021) where iron-reducers are active. We address the research questions: how the time-dependent concentrations of LMM thiols relate to growth state of cells; if thiol turnover is responding to the supply of essential trace metals [Fe(II)) and/or the exposure to toxic trace metals (Hg(II)]; how the metabolic turnover of thiols is perturbed by exposure to exogenous thiols.

## Results and discussion

**Figure 1:**
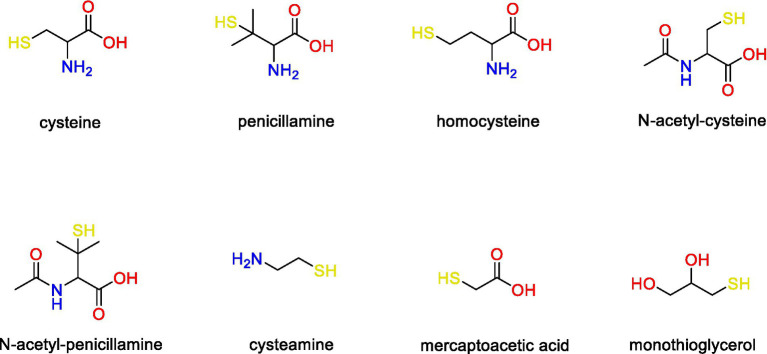
Chemical structure of the LMM thiol compounds included in this study.

In this study, we focused on the eight LMM thiol compounds cysteine, penicillamine, homocysteine, N-acetyl-cysteine, N-acetyl-penicillamine, cysteamine, mercaptoacetic acid and monothioglycerol (Figure 1) of which the first six are structurally related to cysteine. These compounds have previously been detected intracellularly and in the extracellular medium of *G. sulfurreducens* assays (Adediran et al., 2019) using a mass spectrometry-based method (detection limit ~0.1 to 0.5 nM; Liem-Nguyen et al., 2015) The compounds have further been found in appreciable concentrations in natural biofilms (Leclerc et al., 2015; Bouchet et al., 2018) and soil porewater (Van et al., 2021), which are important natural habitats for iron-reducing bacteria.

### Time-dependent concentrations of LMM thiols related to cell growth state

**Figure 2:**
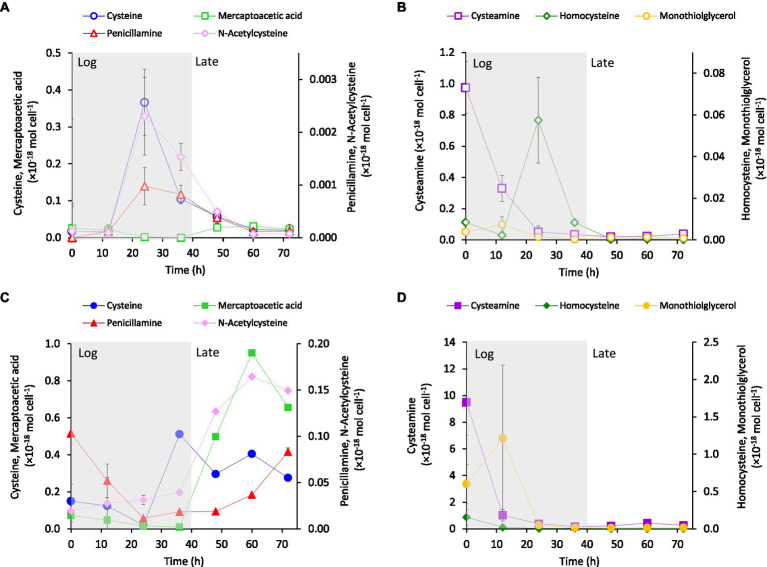
Average concentrations normalized to cell density (mol cell^−1^) of cysteine-related LMM thiols **(A,B)** intracellular and **(C,D)** in the extracellular growth medium over time at addition of 3.7 μM Fe(II) to the growth medium. The main growth phases: exponential growth (Log) and late exponential-stationary growth (Late) are indicated by shading in the figure (see Supplementary Figure S1A for growth curves). Error bars represent standard error, *n* = 4 and are smaller than visible in most cases.

The intracellular (Figures 2A,B) and extracellular (Figures 2C,D) concentrations of LMM thiols were measured during cell growth of *G. sulfurreducens*. The thiols were separated into two groups depending on their concentration-time profiles in the extracellular medium. One group showed increasing concentrations during the late growth phase (Figure 2C) and one group showed highest concentration during the log phase (Figure 2D). The intracellular concentrations expressed per cell (mol cell^−1^) for cysteine, homocysteine, penicillamine and N-acetyl-cysteine showed a transient profile with maximum concentrations in the mid-log phase (~20–30 h; Figures 2A,B), although the concentrations of the two latter compounds were low. Cysteamine showed an exponentially decreasing concentration pattern while the concentrations of mercaptoacetic acid and monothioglycerol remained comparably stable at low concentrations. The concentration of N-acetyl-penicillamine was close to or below detection limit in all samples. In the extracellular medium, both per cell (Figure 2C) and molar (Supplementary Figure S1) concentrations of cysteine, penicillamine, mercaptoacetic acid and N-acetyl-cysteine increased systematically during the late log-phase of cell growth. The per cell concentrations of cysteamine, homocysteine and monothioglycerol decreased systematically over time (Figure 2D) while the molar concentrations were fairly constant or varied irregularly (Supplementary Figure S1). The results in Figure 2 and Supplementary Figure S1 suggest that cysteine, penicillamine and N-acetyl-cysteine were synthesized in excess of cellular demand and incorporation from the mid-log phase, with partial export as a consequence. Also homocysteine was produced intracellularly (Figure 2B) but the extracellular concentrations were remarkably stable at concentrations close to detection limit throughout growth (Figure 2D). This suggests a tight coupling between homocysteine production and utilization by the cells, reducing intracellular accumulation and export/leakage out of the cell for this compound. The low intracellular concentration in the log phase and high extracellular concentration in the late phase of mercaptoacetic acid suggest that this compound is largely a secondary metabolite. The substantial decrease in both intracellular and extracellular per cell concentrations of cysteamine, and to a lesser extent monothioglycerol, were related to the increased cell density as the molar concentration of cysteamine remained high without a clear temporal pattern during the cell growth cycle (Supplementary Figure S1). Overall, the thiol concentrations were lower intracellular than extracellular and the concentration profiles differed distinctly among the thiol compounds (Figure 2). These observations demonstrated that cell lysis was not a major process causing the extracellular accumulation of thiols.

### Thiol turnover in response to nutritional Fe(II) addition

**Figure 3:**
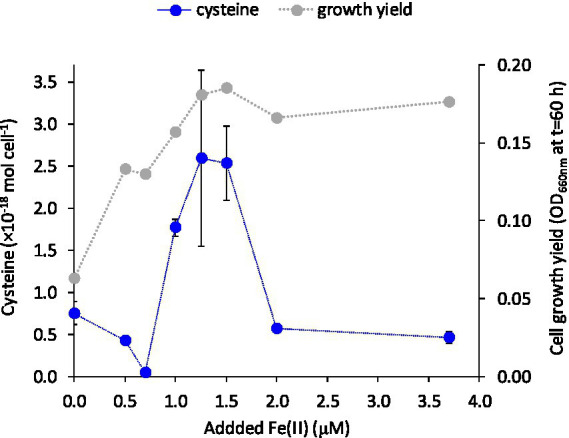
Cysteine concentration (mol cell^−1^) in the extracellular medium at 60 h and final cell yield (OD_660_ at 60 h) versus added Fe(II) concentration. Error bars represent standard error, *n* = 3 and are smaller than visible in several cases.

We investigated if the turnover and release of LMM thiols to the extracellular medium were responding to the supply of the essential trace metal Fe(II). Cells of *G. sulfurreducens* are normally grown with 3.7 μM of the micronutrient Fe(II) in the medium (c.f. Figure 2) as recommended by the supplier (DSMZ, Braunschweig, Germany; Caccavo et al., 1994). To examine if thiol turnover was responding to Fe(II) supply, cells were grown in the presence of variable additions of Fe(II) (0–3.7 μM Fe). Growth rate decreased by 50 and 20% when cells were grown in medium amended with 0 and 0.5 μM Fe(II), respectively, relative to medium with higher Fe(II) levels. Growth yield (OD at full growth, *t* = 60 h) increased with added Fe(II) concentration up to 1.25 μM and then remained fairly unaffected at further increased Fe(II) amendments (Figure 3; Supplementary Figure S2). The concentrations of cysteine, and to some extent also penicillamine, in the medium were highly dependent on the supplied Fe(II) concentration with a “bell-shaped” response in cysteine and penicillamine concentrations which reached a maximum (2.5 × 10^−18^ mol cell^−1^ in the stationary phase at 60 h for cysteine) at intermediate added Fe(II) concentrations of 1.25–1.5 μM (Figure 3; Supplementary Figure S2). Interestingly, this Fe concentration (1.25–1.5 μM) corresponds to the minimum Fe(II) concentration sufficient to alleviate Fe limitation on growth. Concentrations of the other LMM thiol compounds showed no systematic relationship with Fe(II) concentration. These results suggest that the production and export of cysteine (and possibly penicillamine) by *G. sulfurreducens* is at least partly linked to iron homeostasis.

**Figure 4:**
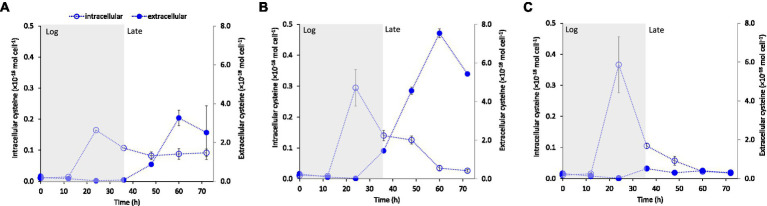
Intracellular and extracellular cysteine concentration (mol cell^−1^) over time of growing *G. sulfurreducens* cells at added Fe(II) concentrations to the medium of **(A)** 0.5 μM, **(B)** 1.5 μM and **(C)** 3.7 μM. The main growth stages: exponential growth (Log) and late exponential-stationary growth (Late) are indicated by shading in the figure (see Supplementary Figure S3 for growth curves). Error bars represent standard error, *n* = 3 and are smaller than visible in several cases.

To further explore the potential relationship between cysteine production and export and Fe(II) supply, we measured intracellular and extracellular LMM thiol concentrations at variable Fe(II) concentrations in separate, but identical experiments. Independent of starting Fe(II) concentration, intracellular cysteine levels consistently peaked in the mid log growth phase (24 h) with a subsequent decline during late-exponential and stationary growth (35–60 h; Figure 4). This decline in intracellular cysteine coincided with a rise in extracellular cysteine concentration after 35 h, which was considerably enhanced at Fe(II) deficient conditions of 0.5 and 1.5 μM Fe(II) (Figures 4A, B, respectively) compared to conditions with sufficient Fe(II) (3.7 μM Fe(II), Figure 4C). These results suggest an increased excess of synthesized cysteine when Fe(II) concentration is near the boundary between Fe-starvation and Fe-sufficiency (0.5–1.5 μM) and as a result, higher concentrations of cysteine accumulate outside the cell during late growth.

Previous transcriptomic studies in *G. sulfurreducens* have shown that Fe limitation leads to a 5-fold upregulation of genes involved in cysteine biosynthesis (*cysE*, GSU2572) and transfer of sulfur to Fe-S clusters (cysteine desulfurase, GSU2570; Embree et al., 2014). The results in Figure 4 thus suggest that the link between Fe(II) and cysteine may be governed by Fe trafficking and allocation to such Fe-proteins in the periplasm and surface proteins. The genome of *G. sulfurreducens* contains homologs (GSU1215 and GSU1216) to CydDC (Pittman et al., 2002), an ABC exporter important for the transport of cysteine to the periplasm for cytochrome assembly. *G. sulfurreducens* has a high abundance of cytochromes in the periplasm and at the cell surface (Ding et al., 2006), and it is possible that even slight Fe deficiency suppresses the assembly of cytochromes and Fe-S clusters, leading to the accumulation of excess cysteine in the periplasm and subsequent leakage out of the cell.

It is less likely that the release of cysteine to the extracellular medium is part of a strategy by *G. sulfurreducens* to acquire Fe(II) during cell growth, analogous to the release of siderophores (Neilands, 1981; Khan et al., 2018). It is known that *G. sulfurreducens* acquires Fe(II) *via* the ferrous iron transport system (Feo) where Fe(II) is actively transported from the periplasm to the cytosol after its presumed diffusion into the periplasm *via* undefined porins (Cartron et al., 2006; Lau et al., 2016; Sestok et al., 2018). In low sulfide systems, uncomplexed Fe(II) is quite soluble at circumneutral pH, decreasing the need for metal-binding ligands to facilitate uptake. However, LMM thiols do chemically react with a variety of trace metals, including Fe(II), and thus, are likely to influence the chemical speciation and bioavailability of trace metals in the environment.

### Thiol turnover in response to Hg(II) exposure

Several studies on contrasting bacteria have shown that exposure to toxic metals can cause responses in the expression of genes for thiol synthesis and in the concentrations of specific thiol compounds (Gao et al., 2013; Rodriguez-Rojas et al., 2016; Norambuena et al., 2018; Dulay et al., 2020). We investigated whether *G. sulfurreducens* likewise produced thiols in response to exposure to the toxic metal Hg(II). Because Hg(II) has a very high affinity to thiol compounds, production and cellular release of such compounds could potentially be an efficient approach to sequester Hg(II) and alleviate exposure. Adediran et al. (2019) previously showed that Hg(II) exposure to *G. sulfurreducens* did not impact the concentration levels of extracellular thiols measured after 48 h. However, it is possible that a transient response in extracellular thiol concentrations might be evident at shorter times not visible after 48 h. Transient responses in thiol production have previously been observed for *Pseudomonas* spp. exposed to Hg (Rodriguez-Rojas et al., 2016).

We examined the release of LMM thiols in washed cell assays containing 0–200 nM Hg(II). Sublethal exposure concentrations in the nM range are commonly used in recent studies to investigate transcriptomic and metabolomic responses to Hg exposure in microorganisms, e.g., (Beauvais-Fluck et al., 2017, 2018; Slaveykova et al., 2021). Washed cell assays were used for this experiment to remove metabolites released during growth and better control the chemical speciation of Hg during the initial time points following Hg exposure. Acetate (electron donor) and fumarate (electron acceptor) were added to the washed cell assay buffers to sustain metabolically active cells although the concentrations were lower compared to the growth medium (Supplementary Table S1). The concentration of the eight LMM thiols in the extracellular assay buffer were initially higher in assays containing Hg than control assays immediately after inoculation (*t* = 0 h, Supplementary Figure S4). However, from 2 h onwards the concentration of LMM thiols were very similar in all assays. These results show that Hg(II) additions in the concentration range 0–200 nM did not impact the concentration levels or time dynamics of extracellular LMM thiols in *G. sulfurreducens* cell assays within the first 4 h, nor after longer time intervals, in agreement with Adediran et al. (2019). Our results therefore do not support the hypothesis that thiol export would be part of a defense mechanism against Hg(II) exposure.

An important observation between this study and the previous study by Adediran et al. is the remarkable reproducibility in the pattern of LMM thiols released by washed cell assays of *G. sulfurreducens*. The sum concentration of LMM thiols were nearly identical and the concentration of individual thiol compounds agreed within a factor of two across the two studies (except for cysteamine). For example, after 6 h and 48 h respectively, the sum concentration of the eight LMM thiols were (0.90 ± 0.40) × 10^−18^ mol cell^−1^ and (1.1 ± 0.2) × 10^−18^ mol cell^−1^ in our study compared to (1.0 ± 0.05) × 10^−18^ mol cell^−1^ and (1.2 ± 0.05) × 10^−18^ mol cell^−1^ in Adediran et al., respectively.

Several previous studies have reported responses in thiol production and concentrations in bacteria exposed to toxic metals, including Hg. Up-regulation of several thiol synthesis pathways in response to metal exposure was reported, including cysteine synthase in *E. coli* exposed to Hg (Gao et al., 2013; Norambuena et al., 2018). The observed upregulated thiol synthesis genes were, however, often accompanied with decreased concentrations of free thiol compounds likely due to metal binding as part of an internal metal sequestration mechanism (Rodriguez-Rojas et al., 2016; Norambuena et al., 2018; Dulay et al., 2020). Also secretion of thiols binding toxic metals extracellular and alleviating cellular uptake have been reported as a bacterial defense mechanism (Mahbub et al., 2017; Meesungnoen et al., 2021). While *G. sulfurreducens* has the capacity to take up Hg(II) when complexed to some LMM thiols, but not others (Schaefer et al., 2011), our results suggest that exposure to Hg(II) does not appear to alter the distribution of LMM thiols released outside the cell.

### Perturbation of thiol turnover by external loadings of cysteine

As briefly discussed in the Introduction, in its natural habitat *G. sulfurreducens* is likely to be exposed to exogenous thiols produced by other microorganisms and in laboratory experiments thiols have been added to *G. sulfurreducens* assays for several purposes. Little is known about the impact (including time-dynamics) of external thiol exposure of *G. sulfurreducens*. In this study, we added cysteine to investigate potential perturbations in LMM thiol turnover by *G. sulfurreducens* in response to external thiol loadings. We selected cysteine because it is a predominant LMM thiol in natural biofilms (Leclerc et al., 2015; Bouchet et al., 2018) and is thus environmentally relevant. Further, as shown in Figure 2, cysteine is a predominant LMM thiol produced by *G. sulfurreducens* and is thus not expected to induce anomalous metabolic responses.

**Figure 5:**
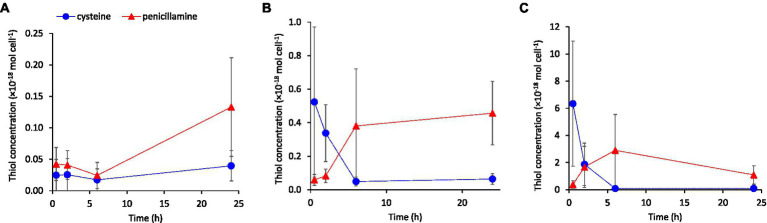
Extracellular concentrations of cysteine and penicillamine over time in washed-cell assays with “Standard buffer” containing 30 nM Hg(II) following the addition of **(A)** 0, **(B)** 100 nM or **(C)** 600 nM cysteine. Error bars represent standard error, *n* = 3.

We added cysteine in the concentration range 100 to ~1,000 nM corresponding to typical concentrations reported for soil pore waters (Van et al., 2021) and biofilms (Leclerc et al., 2015; Bouchet et al., 2018), which are important natural habitats for iron-reducing bacteria. These experiments were also part of a study investigating the effect of cysteine addition on the formation of methylmercury (manuscript in preparation) and therefore 30 nM Hg(II) was also added to the assays. We used three types of *G. sulfurreducens* washed-cell assays with increasing nutrient content: “Standard buffer,” “Metabolite buffer,” and “Nutrient buffer” as described in Supplementary Table S1. Cysteine was added either as a pure standard solution or as a component of the Metabolite buffer which contained metabolites produced and excreted by *G. sulfurreducens* during exponential cell growth (see Materials & Method). A consistent result in all exposure experiments with exogenous cysteine was a decrease in cysteine and increase in penicillamine concentration over time in the extracellular medium (Figure 5; Supplementary Figure S5). This shift from cysteine to penicillamine over time was amplified at increased addition of cysteine, i.e., the shift occurred at earlier time points and the penicillamine/cysteine concentration ratio at extended times increased. Indeed, after a time point between <2 to 6 h of incubation, the addition of cysteine resulted in higher penicillamine than cysteine concentration in the medium for most treatments. For the experiments with 100 or 600 nM cysteine additions, the concentration of penicillamine at 6 or 24 h corresponded to 20 to 65% of the initially added cysteine concentration. The decrease in cysteine was particularly rapid in the “Nutrient buffer” assay (Supplementary Figure S5), which corresponded to the highest cell growth rate in these experiments. None of the other LMM thiols appeared to be systematically affected by the cysteine additions (data not shown).

**Figure 6:**
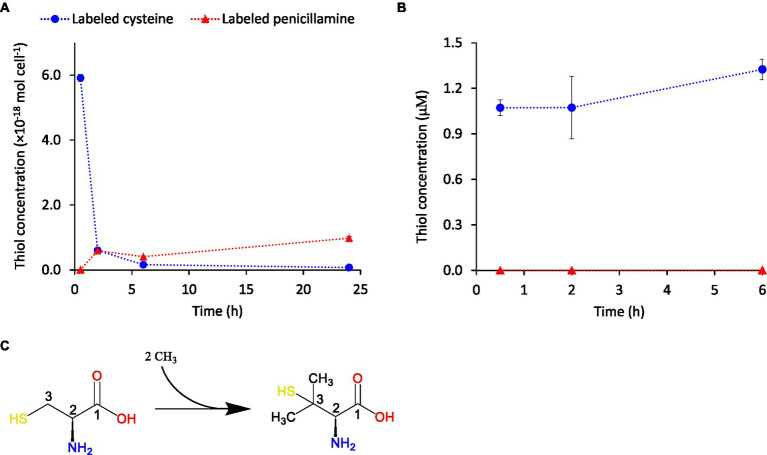
Concentrations over time of isotopically labeled cysteine (blue circles) and penicillamine (red triangles) in extracellular assay buffer following the addition of 1.0 μM of isotopically labeled cysteine (L-Cysteine-^13^C_3_,^15^N) in **(A)** the presence of *G. sulfurreducens* cells in “Standard buffer” and in **(B)** the absence of cells but in the presence of extracellular metabolites isolated from separate cell incubation assays. Error bars represent standard error, *n* = 3. **(C)** Reaction scheme illustrating the dimethylation of the C-3 atom of cysteine leading to the formation of penicillamine.

To further investigate if the turnover of cysteine and penicillamine was a consequence of formation of penicillamine with cysteine as a direct substrate, or a consequence of indirect metabolic responses, we added isotopically enriched cysteine (L-Cysteine-^13^C_3_,^15^N. Linear formula: HS^13^CH_2_^13^CH(^15^NH_2_)^13^COOH) to *G. sulfurreducens* assays. No Hg(II) was added in these experiments. The corresponding isotopically enriched penicillamine compound (HS^13^C(CH_3_)_2_^13^CH(^15^NH_2_)^13^COOH) was detected in appreciable concentrations in the medium (Figure 6A; Supplementary Figure S6), which confirmed that penicillamine was formed with cysteine as a direct substrate. The detected penicillamine corresponded to ~15% of the added cysteine and was thus lower in these experiments compared to the ones in Figure 5. However, the addition of isotopically labeled cysteine did not cause any increase in non-labelled penicillamine which is a further strong support for direct formation of penicillamine from cysteine. Isotopically labeled cysteine and penicillamine were not detected in controls without added L-Cysteine-^13^C_3_,^15^N (Supplementary Figure S7). To verify that penicillamine was formed by bacterial cells and not in the dissolved assay buffer we carried out separate control experiments with buffer only. These control assays were based on assay buffer isolated from washed *G. sulfurreducens* assays after 24 h. In this way the buffer also contained metabolites excreted by cells. Cells were removed by filtration and 1 μM isotopically labeled cysteine was added and the samples incubated up to 6 h. No isotopically labeled penicillamine was detected in these control assays (Figure 6B) which showed that its formation did not occur in the absence of cells and was not mediated by constituents of the assay buffer or extracellular metabolites. We further measured intracellular thiol concentrations in cells exposed to isotopically labeled cysteine. In these samples, isotopically labeled cysteine was detected at 30 min but not at later time points (Supplementary Figure S8). Low signals were observed for isotopically labeled penicillamine but concentrations were below quantification limit. Taken together, these results suggest that externally added cysteine was methylated to penicillamine either at the cell surface or after internalization of cysteine with a subsequent rapid export of the formed penicillamine from the cells. Either of these two cases would explain why there was no intracellular build-up of isotopically labeled cysteine or penicillamine while cysteine gradually decreased and penicillamine increased in the extracellular medium. Interestingly, the intracellular concentration of endogenous cysteine (i.e., produced by the cells) appeared unaffected by exogenous additions of isotopically labeled cysteine and remained at a fairly constant concentration level (Supplementary Figure S8). Also isotopically enriched N-acetylcysteine (HS^13^CH_2_^13^CH(^15^NH)COCH_3_^13^COOH) was detected at low concentrations in the assay buffer showing a direct formation also of N-acetylcysteine from cysteine in *G. sulfurreducens*.

To the best of our knowledge biotic *de novo* formation of penicillamine has not previously been reported. Major thiols commonly found in prokaryote and eukaryote organisms include coenzyme A, coenzyme M, trypanothione, mycothiol, ergothioneine, and the ovothiols (Fahey, 2001; Hand and Honek, 2005; Wang et al., 2015). In addition to these, several cysteine-containing peptides have been identified, e.g., glutathione, arginine-cysteine and glutamine-cysteine (Dupont et al., 2004). Penicillamine is a very important thiol compound in clinical contexts as it is a major degradation product in penicillin treatment (Abraham et al., 1943; Perry et al., 1965) and because penicillamine has been used as an active drug substance in a range of clinical treatments [e.g., for Wilson’s disease (Pugliese et al., 2021)]. The literature on pharmacokinetics, side-effects and degradation of penicillamine is consequently vast. The compound has been less studied in microbial organisms but it is not generally utilized as a source of energy or to synthesize biomolecules by microbes, although at least one exception has been reported (Yanagidani et al., 1999). Notably, two studies have recently reported the presence of penicillamine in oxygen-deficient natural environments. Bouchet et al. (2018) reported appreciable concentrations in tropical lake biofilms and periphyton and Van et al. (Van et al., 2021) sporadically detected penicillamine in boreal wetland porewaters. The origin of penicillamine in these environments is unknown but our findings suggest that it might originate from biosynthesis by anaerobe microbes and open for a reevaluation of the metabolic roles of this widely studied thiol compound.

Previous studies have demonstrated that penicillamine is less “reactive” than cysteine with respect to several contrasting biological responses (Boyde, 1968; Friedman, 1977), which has been explained by the more sterically hindered thiol group in penicillamine. We further tested the reactivity of penicillamine in *G. sulfurreducens* assays by adding 500 nM penicillamine to the external medium. In sharp contrast to the experiments with added cysteine (Figures 5, 6), the concentration of penicillamine in the external medium remained reasonably stable and did not systematically decrease over time (Supplementary Figures S9A,B). Further, despite the high extracellular concentration of 500 nM, practically no penicillamine was detected intracellular in these experiments (Supplementary Figure S9C). These results show that penicillamine was not internalized or metabolized by *G. sulfurreducens* under the experimental assay conditions used. Formation of penicillamine could thus be part of a strategy by the organism to maintain cysteine homeostasis when exposed to high concentrations of exogenous cysteine. While cysteine is an essential amino acid with multiple critical biological functions, even low concentrations of excess intracellular cysteine can cause a range of toxic effects including to promote the Fenton reaction and generate damaging hydroxyl radicals (Carlsson et al., 1979; Park and Imlay, 2003; Turnbull and Surette, 2010; Reitzer, 2015). Cells therefore tightly regulate cysteine homeostasis and maintain a low intracellular concentration by coordinating cysteine biosynthesis, utilization, oxidation, and transport (Loddeke et al., 2017). It has been reported that the homeostasis level of intracellular cysteine in growing *E. coli* is 100 to 200 μM (Park and Imlay, 2003). Both homeostasis and toxic intracellular cysteine concentrations are, however, uncertain for *G. sulfurreducens*. The intracellular cysteine concentrations measured in our study without added exogenous cysteine corresponded to 10 to 200 μM (both for cell growth and washed-cell assay conditions), assuming a cell volume (Caccavo et al., 1994) of 2 × 10^−15^ l cell^−1^. This cysteine concentration range is comparable to the levels reported for *E. coli* (Park and Imlay, 2003). At external addition to the assay buffer of 1 μM isotopically labeled cysteine (Supplementary Figure S8), the intracellular concentration of labeled cysteine reached a maximum of ~1,000 μM at 30 min and was not detectable at longer incubation times (as discussed above) while native cysteine remained at ~25 to 300 μM. These results suggest that *G. sulfurreducens* tightly regulates the intracellular cysteine homeostasis at concentrations not exceeding 200–300 μM. It remains to be demonstrated, however, if methylation of cysteine to penicillamine is a mechanism for intentional inactivation and alleviation of excess cysteine by *G. sulfurreducens* and if the process is widespread among bacteria and/or other organisms.

The mechanism for dimethylation of the cysteine C-3 atom is unclear. The methylation of *sp*^3^-hybridized carbon atoms (like the C-3 atom in cysteine) generally requires an activation reaction as there are no free electrons available for a nucleophilic attack. Certain types of radical *S*-adenosylmethionine (SAM) enzymes are capable of methylating *sp*^3^-hybridized carbon atoms *via* the production of a carbon radical in the substrate molecule followed by a nucleophilic attack of the C-radical, or of a subsequently formed carbanion, onto a methylcobalamin (Bauerle et al., 2015). A well-known example of such reactions is the C-methylation of arginine and glutamine in methyl-coenzyme M reductase (Ermler et al., 1997; a central enzyme in methanogenesis) for which a complete reaction mechanism was recently proposed (Fyfe et al., 2022). It remains to be identified if SAM enzymes are also responsible for the methylation of cysteine to penicillamine in *G. sulfurreducens*.

## Conclusion

Much remains to be learned about metabolic turnover of thiols, including cysteine-related ones, in general and for anaerobic microorganisms in particular. Our study reveals several novel insights regarding environmentally relevant nanomolar concentrations of such compounds in *G. sulfurreducens*. Specifically, our results point to a link between cysteine and iron homeostasis pathways. This could include strategies for Fe(II) complexation and uptake and/or cellular distribution and allocation of Fe(II) to iron protein. Our study is further the first to report metabolic synthesis of penicillamine. The results suggest that penicillamine is formed by the transfer of two methyl groups to the C-3 atom of cysteine by *G. sulfurreducens* cells and that the formed penicillamine is released from the cell. The fact that penicillamine formation increased with external exposure to cysteine may suggest that its formation is a part of the metabolic strategy to maintain a low intracellular cysteine concentration. The mechanisms for methylation of cysteine to penicillamine remain to be identified. This study opens up new avenues of research into the metabolic roles of penicillamine and calls for more attention to this compound in studies of microorganisms. Overall, *G. sulfurreducens* maintains the homeostasis of cysteine at intracellular concentrations not exceeding 200–300 μM by regulating its intracellular synthesis, cellular export and methylation to penicillamine in response to external supply of Fe(II) and cysteine. Endogenous cysteine levels are mainly regulated by cysteine production and cellular export/translocation. In the presence of excess exogenous cysteine, however, these mechanisms are likely not sufficient to maintain homeostasis and the biomethylation of cysteine to the less reactive compound penicillamine (which subsequently is released from cells) is upregulated.

## Material and methods

*Geobacter sulfurreducens* PCA (ATCC 51573, purchased from DSMZ, Braunschweig, Germany; Caccavo et al., 1994) was grown under N_2_ atmosphere in MOPS-buffered growth medium at 30°C and pH 6.8 using acetate (10 mM) as electron donor and fumarate (30 mM) as electron acceptor as described by Schaefer and Morel (2009). All media and buffers were prepared in acid-clean serum bottles, flushed with N_2_, sealed with Teflon stoppers and autoclaved. The medium pH was 6.8–6.85 in all experiments. All experiments were repeated at least in triplicates and carried out at 30°C in the dark under N_2_ atmosphere in a glovebox. In all experiments the cell density was monitored during incubation by optical density measurements at λ = 660 nm (OD_660_) on an UV-1201 spectrophotometer, Shimadzu.

### Turnover of LMM-thiol compounds during cellular growth phase

LMM-thiol production was studied during the cell growth after bacterial inoculation (2% (v/v)) of a late-exponential-phase growth culture into fresh growth medium (Supplementary Table S1). The turnover of LMM-RSH in growth medium was studied at Fe(II) concentrations from 0 to 3.7 μM using low Fe growth medium or growth medium (Supplementary Table S1) after bacterial inoculation (2% (v/v)) of a late exponential-phase growth culture (grown in 3.7 μM Fe). Samples were collected for extracellular and intracellular LMM-thiol determinations at multiple time points between inoculation and stationary phase and processed for LMM-RSH analyzes as described below.

### Turnover of LMM-thiol compounds in washed-cell assay conditions

For the washed-cell assay experiments, cells were grown to mid exponential phase (OD_660nm_ = 0.12–0.18) in growth medium, harvested by centrifugation (4,000 g for 8 min, 5°C), washed twice with carbon free assay medium, centrifuged and resuspended in different assay buffers with increasing nutrient content was studied:” Standard buffer,” “Metabolite buffer,” and “Nutrient buffer” (Supplementary Table S1). Acetate (1 mM) and fumarate (1 mM) were added to all assays albeit at lower concentrations compared to the growth medium. The” Standard buffer” was prepared as described in Schaefer and Morel (2009). The “Metabolite buffer” was prepared on the day of experiments by filtering growth culture of *G. sulfurreducens* under Fe(II) depleted condition (1.5 μM) in the late exponential growth phase (OD_660nm_ = 0.15–0.2) through a 0.2 μm syringe filters. The filtrate was added (10% (v/v)) to the “Standard buffer” to generate the “Metabolite buffer” assays. The “Nutrient buffer” assay was obtained by adding 10% (v/v) Growth medium to standard buffer.

All washed-cell assays were pre-equilibrated with respect to the chemical components of each buffer for 1 h at 30°C incubation temperature under dark condition prior to inoculation with 1 ml aliquots of washed cell suspension to a final cell density of ~10^8^ cells mL^−1^ (corresponding to an OD_660nm_ of ~0.02; Schaefer and Morel, 2009). The redox state of the bacteria assays was monitored by the redox indicator resazurin. After the addition of cells, the color change of resazurin from pink to clear was monitored and only sufficiently reduced vials (clear) were used. The impact of Hg(II) additions on the concentrations of extracellular LMM-thiol compounds was studied in in Standard buffer assays after equilibration for 1 h with 0 to 200 nM Hg(II) prior to cell addition. Subsamples for extracellular LMM-thiol analyzes were collected at different time points up to 48 h and processed as described below.

### Perturbation of thiol turnover by addition of exogenous cysteine and penicillamine

The turnover of LMM-thiol compounds was studied in washed-cell assays at different external loading of cysteine and penicillamine. Cell assays were prepared with the three different buffers described above. Prior to cell addition, all assays were equilibrated for 1 h in the dark at 30°C with 0, 100, 600, or 1,000 nM cysteine (L-Cysteine (≥97%), Sigma Aldrich). These experiments were also part of a study investigating the effect of cysteine addition on the formation of methylmercury (manuscript in preparation) and therefore also 30 nM Hg(II) was added to the assays prior to equilibration. Experiments with 1,000 nM isotopically labeled L-cysteine (L-Cysteine-^13^C_3_,^15^N; Linear formula: HS^13^CH_2_^13^CH(^15^NH_2_)^13^COOH, Sigma Aldrich) and 500 nM penicillamine were prepared in the same manner in the “Standard buffer.” No Hg(II) was added to these assays. A control was prepared without addition of LMM-thiols. Samples for the determination of extracellular and intracellular LMM-thiols were collected at different time points (0.5, 2, 6 and 24 h) after incubation with *G. sulfurreducens* and analyzed as described below. Also a control without cells was prepared by filtering a pure “Standard buffer” assay after 24 h with a 0.2 μm syringe filter. The filtrate was collected, 1 μM isotopically labeled L-cysteine was added and subsamples were collected at different time points for the determination of extracellular LMM-thiols.

### Determination of extracellular and intracellular LMM thiol concentrations

Samples for extracellular and intracellular LMM-RSH analyzes were collected at different time points after incubation with *G. sulfurreducens* in growth medium or washed-cell assay conditions as described above. Subsamples for extracellular LMM-RSH determination were collected and filtered through a 0.2 μm syringe filter. For the determination of intracellular LMM-RSH compounds, cells were collected by centrifugation (4,000 g for 15 min, 5°C), washed twice with carbon free assay buffer, resuspended in deoxygenated Milli-Q-water (>18 MΩ.cm, Merck Millipore), lysed at 80°C for 10 min and filtered through a 0.2 μm syringe filter. The filtrates were derivatized with 4-(hydroxymercuri) benzoate (PHMB, ≥95%, Sigma Aldrich) and the pH was adjusted to 3 with 5 M HCl (Sigma Aldrich) prior to analyzes. The concentration of specific LMM-RSH compounds were determined by liquid chromatography (Agilent Zorbax SB-C8, 2.1 × 100 mm, 3.5 μm) electrospray ionization tandem mass spectrometry (LC-ESI-MS/MS, TSQ Quantum Ultra electrospray ionization triple quadrupole mass spectrometer, Thermo Fisher Scientific, San Jose, CA, United States) after online pre concentration with solid-phase extraction (Waters, Oasis HLB, 2.1 × 20 mm, 15 μm) as described in Liem-Nguyen et al. (2015). No pre-reduction of organic disulfides was performed, thus only the reduced thiol form of each compounds was measured. The derivatization with PHMB stabilizes the thiol compounds and prevents oxidation and thiol compounds are detected in the form of their respective PHMB derivative. Calibration standards were prepared freshly for each run in deoxygenated Milli-Q-water, growth medium or assay buffer and contained the eight LMM thiol compounds. For the experiments with isotopically labeled cysteine (L-Cysteine-^13^C_3_,^15^N, Sigma Aldrich), the monitored masses for each LMM-thiol compound were modified accordingly. All LMM-RSH compounds were purchased from Sigma Aldrich. Individual stock solutions (10 mM) were prepared in deoxygenated MilliQ water for the LMM-RSH compounds: Cysteamine (≥98%), Mercaptoacetic acid (98%), Monothioglycerol (≥97%), L-Cysteine (≥97%), DL-Homocysteine (≥95%), D-Penicillamine (98–101%), N-acetyl-L-cysteine (≥98%), N-Acetyl-L-penicillamine (≥99.0%).Examples of LC-ESI-MS/MS selected reaction monitoring chromatograms for the different LMM thiol compounds in standard solutions and in cell assay samples are given in Supplementary Figures S10, S11, respectively, and the monitored m/z and optimized MS/MS fragmentation parameters are given in Supplementary Table S2.

## Data availability statement

The original contributions presented in the study are included in the article/Supplementary material, further inquiries can be directed to the corresponding author.

## Author contributions

The study was designed and planned by MG, JS, US, and EB. The experimental work was mainly performed by MG. TM performed complementary experimental work. Data analysis was performed by MG, JS, and EB. The first draft of the manuscript was compiled by EB and MG. All authors contributed to the article and approved the submitted version.

## Funding

This work was financially supported by the Swedish Research Council (2017–04537), the Kempe Foundations (SMK-1753 and SMK-1243) and Umeå University. The Swedish Research Council funded project cost, the Kempe Foundations funded instrumentation and Umeå University part of salary cost.

## Conflict of interest

The authors declare that the research was conducted in the absence of any commercial or financial relationships that could be construed as a potential conflict of interest.

## Publisher’s note

All claims expressed in this article are solely those of the authors and do not necessarily represent those of their affiliated organizations, or those of the publisher, the editors and the reviewers. Any product that may be evaluated in this article, or claim that may be made by its manufacturer, is not guaranteed or endorsed by the publisher.
