# Supplementary material for: Metabolic turnover of cysteine-related thiol compounds at environmentally relevant concentrations by Geobacter sulfurreducens
Source: Front Microbiol. 2023 Jan 11;13:1085214. doi: 10.3389/fmicb.2022.1085214 (PMC9874932; doi:10.3389/fmicb.2022.1085214)
Supplement: Supplementary file 1 [file Data_Sheet_1.docx]

**Supplementary Information:**

**Metabolic turnover of cysteine-related thiol compounds at environmentally relevant concentrations by *Geobacter sulfurreducens***

Mareike Gutensohn^1^, Jeffra K. Schaefer^2^, Torben J. Maas^3^, Ulf Skyllberg^4^, Erik Björn^1^*

^1^Department of Chemistry, Umeå University, SE- 90187 Umeå, Sweden

^2^Department of Environmental Sciences, Rutgers University, 14 College Farm Road, New Brunswick, New Jersey 08901, United States

^3^Institute of Inorganic and Analytical Chemistry, University of Münster, Corrensstraße 48, 48149 Münster, Germany

^4^Department of Forest Ecology and Management, Swedish University of Agricultural Sciences, SE-901 83 Umeå, Sweden

**Table S1.** Chemical composition of the different media and buffers used.

| **Compound** | **Unit** | **Growth medium** | **Low Fe Growth medium** | **Standard buffer** | **Nutrient buffer** | **Metabolite buffer** |
| --- | --- | --- | --- | --- | --- | --- |
| **MOPS** | mM | 10 | 10 | 10 | 10 | 10 |
| **NH_4_Cl** | mM | 5.6 | 5.6 | 0.1 | 0.7 | 0.1 |
| **KCl** | mM | 1.3 | 1.3 | 1.3 | 1.3 | 1.3 |
| **CaCl_2_** | mM | 0.008 | 0.008 | - | 0.0008 | 0.8 |
| **NaCl** | mM | 0.17 | 0.17 | 0.17 | 0.17 | 0.17 |
| **MgSO_4_** | mM | 0.12 | 0.12 | 0.15 | 0.15 | 0.15 |
| **NaH_2_PO_4_** | mM | 0.05 | 0.05 | 5 | 4.5 | 4.51-5.00 |
| **Acetate** | mM | 10 | 10 | 1 | 1.9 | 1.0-1.9 |
| **Fumarate** | mM | 30 | 30 | 1 | 3.9 | 1.0-3.9 |
| **Na_2_SeO_3_** | μM | 0.6 | 0.6 | - | 0.06 | 0-0.06 |
| **Resazurin** | µM | 4 | 4 | 4 | 4 | 4 |
| **NTA** | µM | 78.6 | - | - | 7.86 |  |
| **EDTA** | µM | - | 100 |  | - | 0-10 |
| **CoCl_2_** | µM | 4.24 | 4.24 | - | 0.42 | 0-0.42 |
| **CuSO_4_** | µM | 0.04 | 0.04 | - | 0.004 | 0-0.004 |
| **MnCl_2_** | µM | 29.8 | 100 | - | 2.98 | 0-2.98 |
| **AlK(SO4)_2_** | µM | 0.22 | 0.22 | - | 0.02 | 0-0.02 |
| **H_3_BO_3_** | µM | 1.6 | 1.6 | - | 0.16 | 0-0.16 |
| **Na_2_MoO_4_** | µM | 0.42 | 0.42 | - | 0.04 | 0-0.4 |
| **NiCl_2_** | µM | 0.44 | 0.44 | - | 0.04 | 0-0.4 |
| **ZnSO_4_** | µM | 3.48 | 3.48 | - | 0.34 | 0-0.35 |
| **FeSO_4_** | µM | 3.6 | 0-2.5 | - | 0.36 | 0-015 |

**Table S2.** Precursor and product ions (m/z) and the optimized tube lens and collision energy (V) settings for each LMM-RS–PHMB complex with the Triple Quadrupole Mass Spectrometer Instrument (Thermo Scientific TSQ Quantum Ultra) for the eight LMM-RSH compounds: cysteamine, mercaptoacetic acid, monothioglycerol, L-cysteine, DL-homocysteine, D-penicillamine, N-acetyl-L-cysteine and N-acetyl-D-penicillamine.

| **LMM-RSH compound** | **Precursor ion (m/z)** | **Product ions (m/z)** | **Tube lens (V)** | **Optimum collision energy (V)** |
| --- | --- | --- | --- | --- |
| Cysteamine | 399.7 | 323.0; 383.1 | +104 | 17; 10 |
| Mercaptoacetic acid | 412.8 | 121.3; 323.1 | -107 | 24; 13 |
| Monothioglycerol | 428.8 | 323.1; 355.0 | -133 | 13; 19 |
| L-Cysteine | 442.0 | 234.2; 355.1 | -111 | 47; 17 |
| DL-Homocysteine | 455.8 | 311.0; 355.0 | -116 | 30; 18 |
| Penicillamine | 469.7 | 355.0 | -102 | 17 |
| N-acetyl-L-cysteine | 483.9 | 355.0 | -106 | 18 |
| N-acetyl-D-penicillamine | 511.9 | 355.0 | -112 | 18 |

| **(a)** |  |
| --- | --- |
| **** |  |
| **(b)** | **(c)** |
|  | **** |

**Fig. S1.** Average (a) cell density (cells L^-1^) and (b, c) molar concentrations (nM) of cysteine-related LMM thiols in extracellular growth medium over time at 3.7 μM Fe(II) in the growth medium. The main growth phases: exponential growth (Log) and late exponential-stationary growth (Late) are indicated by shading in the figure. Error bars represent standard error, n=4 are smaller than visible in most cases.

| **(a)** |  |
| --- | --- |
| **** |  |
| **(b)** | **(c)** |
| **** | **** |
| **(d)** | **(e)** |
| **** | **** |

**Fig. S2** Time resolved data for (a) cell density of *G. sulfurreducens* cells and concentrations in the extracellular medium of (b) cysteine, (c) penicillamine, (d) cysteamine and (e) N-acetyl-cysteamine at variable Fe(II) concentrations added to the medium. Error bars represent standard error, n=3.

| **(a)** |
| --- |
|  |
| **(b)** |
|  |
| **(c)** |
|  |

**Fig. S3.** Cell growth curves (cell L^-1^) for *G. sulfurreducens* cells at added Fe(II) concentrations to the medium of (a) 0.5 μM, (b) 1.5 μM and (c) 3.7 μM.

**Fig. S4.** Sum concentrations of thiols in the extracellular medium versus time at different concentrations (0, 30, 100 or 200 nM) of added Hg(II).

| **(a)** | **** | **(b)** | **** |
| --- | --- | --- | --- |
| **(c)** | **** | **(d)** | **** |
| **(e)** | **** | **(f)** | **** |
| **(g)** | **** | **(h)** | **** |

**Fig. S5.** Concentrations of cysteine and penicillamine in the extracellular medium over time in *G. sulfurreducens* washed-cell assays containing 30 nM Hg(II) and (a, c, e, g) “Nutrient buffer” or (b, d, f, h) “Metabolite buffer” with addition of (a, b) 0, (c, d) 100 nM, (e, f) 600 nM or (g, h) 1000 nM of cysteine. Note that the “Metabolite buffer” contains additional cysteine (~100-500 nM) produced and excreted by the bacteria. Error bars represent standard error, n=3 (for the 1000 nM cysteine additions n=1).

| **(a)** |
| --- |
|  |
| **(b)** |
|  |

**Fig. S6.** Selected reaction monitoring chromatograms in LC-MS/MS of (a) isotopically labeled L-cysteine-^13^C_3_,^15^N and the (b) correspondingly labeled penicillamine in extracellular samples of *G. sulfurreducens* washed-cell assays after incubation for 6 h with the addition of 1000 nM L-cysteine-^13^C_3_,^15^N. Inserted reaction schemes show the MS/MS fragmentation of isotopically labeled cysteine and penicillamine giving rise to the monitored signals.

| **(a)** | **(b)** |
| --- | --- |
| **** | **** |

**Fig. S7.** Average (a) extracellular and (b) intracellular concentrations over time for native and isotopically labeled cysteine and penicillamine in *G. sulfurreducens* washed-cell assays without external addition of thiols. Error bars represent standard error, n=3

| **(a)** | **(b)** |
| --- | --- |
| **** | **** |
| **(c)** | **(d)** |
| **** | **** |

**Fig. S8.** Intracellular concentrations over time for (a, b) isotopically labeled cysteine and the corresponding labeled penicillamine and for (c, d ) native cysteine and penicillamine in *G. sulfurreducens* washed-cell assays with (a, c) addition of 1000 nM isotopically labeled L-cysteine-^13^C_3_,^15^N and (b, d) controls without addition. Error bars represent standard error, n=3

| **(a)** | **** |
| --- | --- |
| **(b)** | **** |
| **(c)** | **** |

**Fig. S9.** Cysteine and penicillamine concentrations over time given as (a) extracellular molar concentrations, (b) extracellular per cell concentrations and (c) intracellular per cell concentrations following addition of 500 nM penicillamine to *G. sulfurreducens* washed-cell assay buffer. Error bars represent standard error, n=3.

**(a)
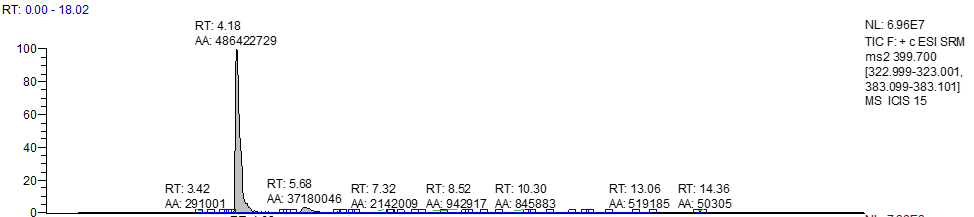
**

**(b)
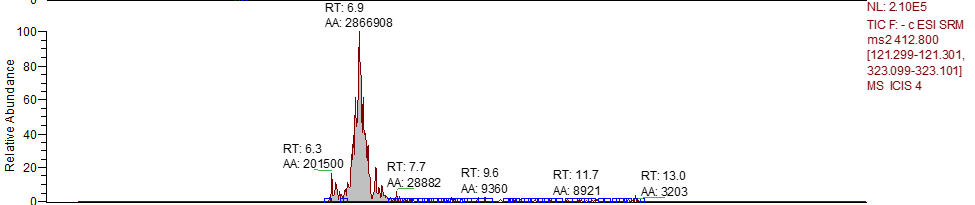
**

**(c)
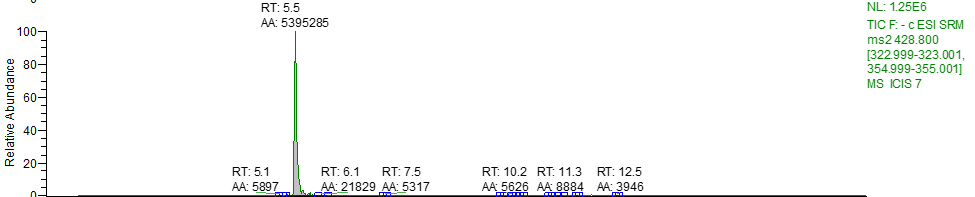
**

**(d)
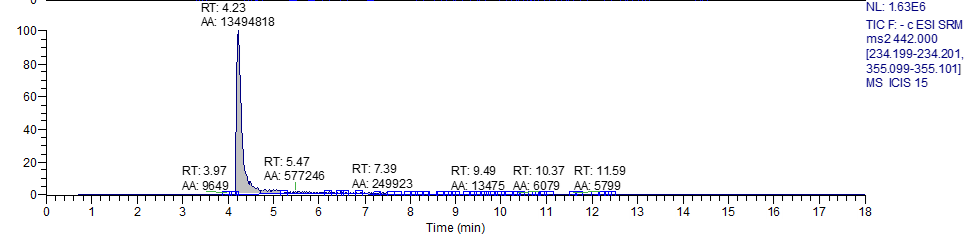
**

**(e)
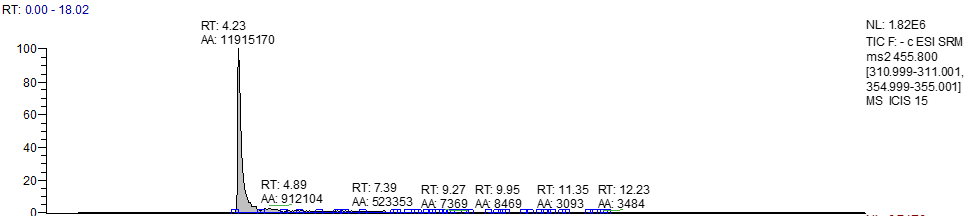
(f)
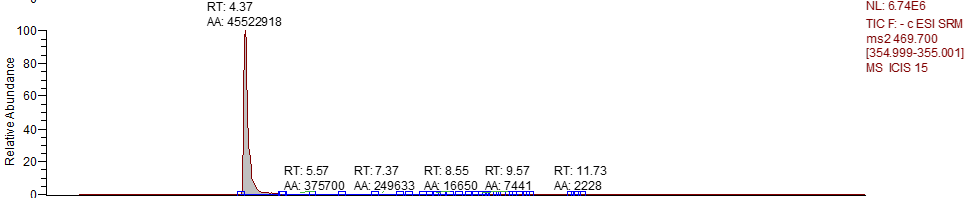
(g)
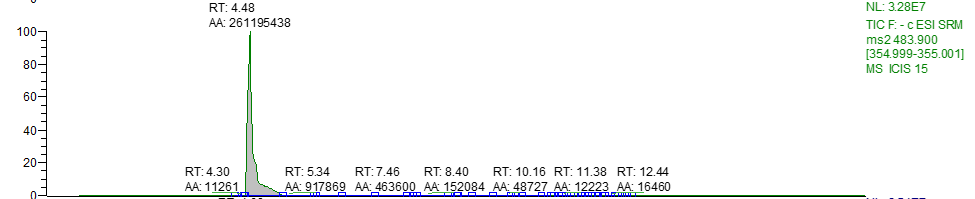
(h)
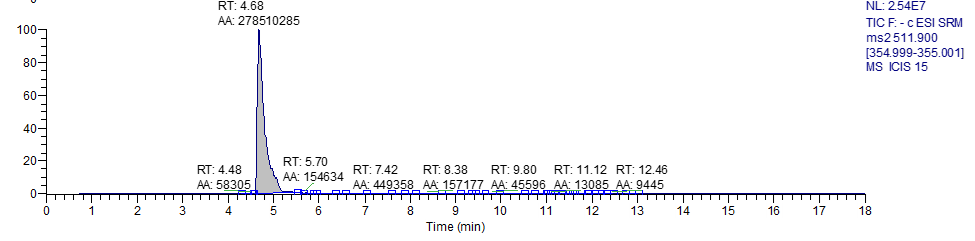
Fig. S10.** Selected reaction monitoring chromatograms in LC-ESI-MS/MS for LMM thiols measured after derivatization with PHMB in standard solutions prepared in assay buffer with 250 nM of each thiol: (a) cysteamine, (b) mercaptoacetic acid, (c) monothioglycerol, (d) L-cysteine, (e) DL-homocysteine, (f) D-penicillamine, (g) N-acetyl-L-cysteine and (h) N-acetyl-D-penicillamine.

**(a)
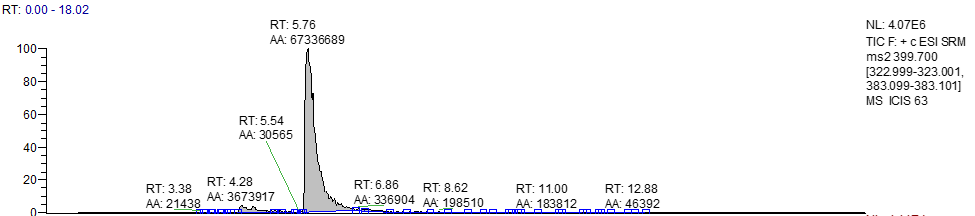
(b)
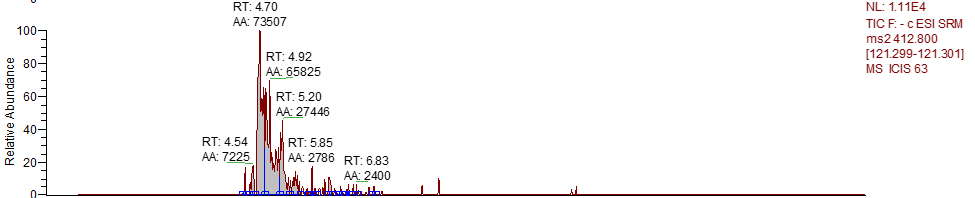
(c)
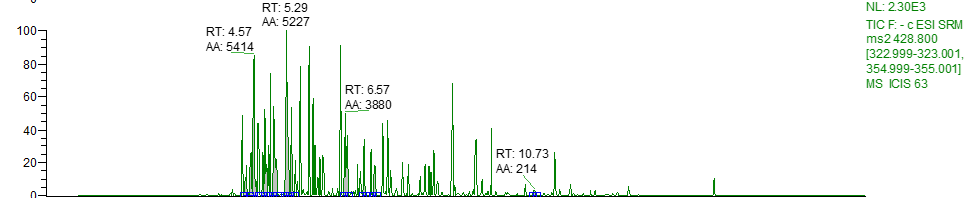
(d)
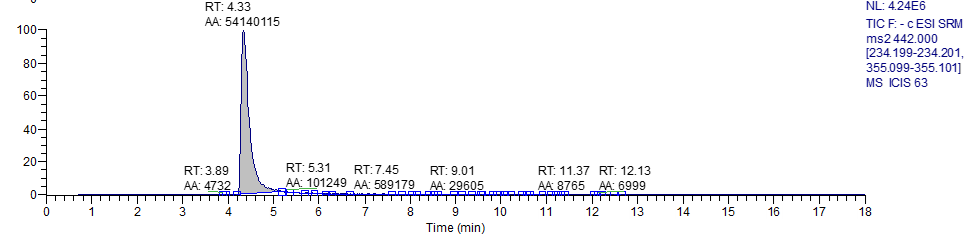
**

**(e)
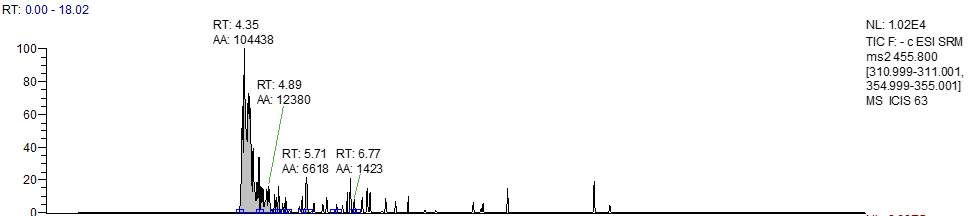
(f)
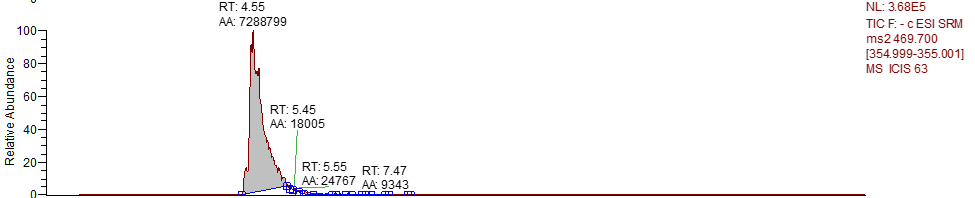
(g)
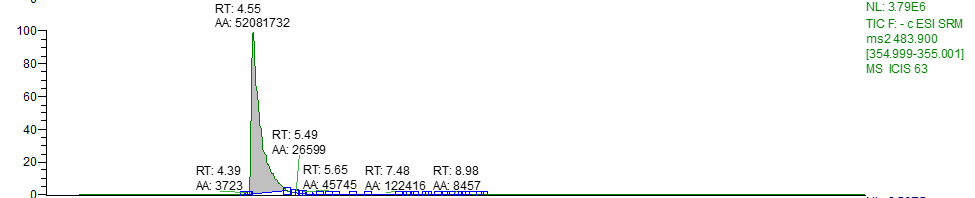
(h)
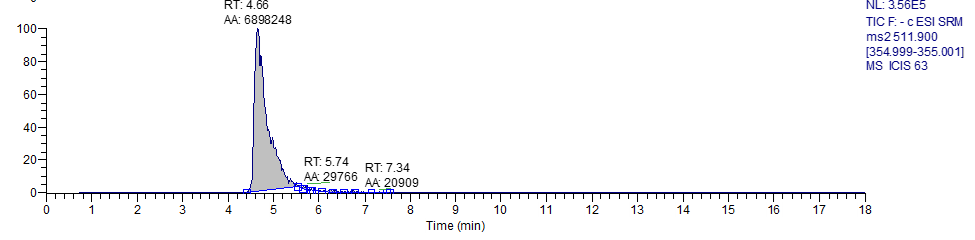
Fig. S11.** Selected reaction monitoring chromatograms in LC-ESI-MS/MS for LMM thiols measured after derivatization with PHMB in extracellular growth medium during cellular growth with 1.5 µM Fe(II): (a) cysteamine, (b) mercaptoacetic acid, (c) monothioglycerol, (d) cysteine, (e) homocysteine, (f) penicillamine, (g) N-acetyl-cysteine and (h) N-acetyl-penicillamine.
